# Relationship between depression and oral microbiome diversity: analysis of NHANES data (2009–2012)

**DOI:** 10.1186/s12903-025-06274-x

**Published:** 2025-06-05

**Authors:** Xichenhui Qiu, Ting Xu, Yiqing Huang, Changning Wei, Lina Wang, Bei Wu

**Affiliations:** 1 Health Science Center, Shenzhen University, No.1066 Xueyuan Avenue, Nanshan District, Shenzhen, 518060 China; 2 School of Nursing, Nanjing Medical University, No.101 Longmian Avenue, Jiangning District, Nanjing, 211166 China; 3 School of Tech X Academy, Shenzhen Polytechnic University, No.7098 Liuxian Avenue, Nanshan District, Shenzhen, 518055 China; 4 School of Medicine, Huzhou Key Laboratory of Precise Prevention and Control of Major Chronic Diseases, Huzhou University, No.1 Xueshi Road, Huzhou, 313000 China; 5 Rory Meyers College of Nursing, New York University, 433 First Avenue, New York, NY 10010 USA

**Keywords:** Depression, Oral microbiome diversity, National health and nutrition examination survey, Cross-sectional study

## Abstract

**Background:**

While the association between the gut microbiome and depression is well studied, the association between the oral microbiome and depression is less well characterized.

**Methods:**

This cross-sectional study analyzed the association between depression and diversity of oral microbiome using data from the 2009–2012 National Health and Nutrition Examination Survey (NHANES). The gene sequencing of 16S ribosomal RNA was adopted for the profiling of oral microbiome. Alpha diversity, quantified by the observed number of amplicon sequence variants (ASVs), and beta diversity, assessed using Bray–Curtis dissimilarity, were evaluated to represent oral microbiome diversity. Depressive symptoms were measured by the Patient Health Questionnaire-9 (PHQ-9) scale, with alpha diversity as the primary predictor. Weighted logistic regression was employed to examine the relationship between depression and microbial alpha diversity. Threshold effect analysis was performed to explore potential nonlinear relationships between the observed ASVs and depression. Subgroup analysis indicated that smoke, excessive alcohol consumption, and oral treatment influenced the association between oral microbiology and depression, with interaction effects observed across gender and racial groups. Beta diversity differences were evaluated using Bray–Curtis dissimilarity and visualized via non-metric multidimensional scaling (NMDS).

**Results:**

A total of 15,018 participants were included, with an average age of 42.25 ± 15.2 years. In the fully adjusted model, the alpha diversity of oral microbiome was significantly negatively correlated with depression (OR = -0.51, 95% CI: -0.79—-0.23, *P* = 0.003). Threshold analysis also revealed a nonlinear association in this relationship, with a significant inflection point as Log10ASVs of 2.32. Furthermore, beta diversity of the oral microbiome differed significantly between the normal and depression groups (*p* = 0.001). Sensitivity analyses showed that the relationship between depression and oral microbial diversity observed in this research was particularly pronounced among non-Hispanic Whites (OR = 0.16, 95% CI: 0.07–0.35) and men (OR = 0.14, 95% CI: 0.06–0.30). Additionally, significant differences in oral microbiome beta diversity were observed between the normal and depression groups (*p* = 0.001).

**Conclusions:**

The findings suggest that the diversity of oral microbiome is negatively correlated with depressive symptoms. Hence, oral dysbiosis may serve as a therapeutic target or biomarker of depression. However, the underlying mechanisms require further investigation.

**Supplementary Information:**

The online version contains supplementary material available at 10.1186/s12903-025-06274-x.

## Background

According to Malhi and Mann, depression, a prevalent and serious chronic mental disorder, is one of the major contributors to global disease [1]. The global prevalence of depression is reported to range from 2 to 6%, varying by region and demographic factors [2]. In 2020, an estimated 264 million people worldwide were affected by depression, a number projected to rise in the coming decades [3]. Depression significantly impairs daily functioning. To develop efficient treatment and prevention strategies, it is essential to understand the underlying mechanisms and contributing factors to depression. However, due to its complexity, the pathogenic mechanisms of depression remain incompletely understood [1].

In recent years, the association between the human microbiome and depression has attracted increasing attention. In the past ten years, research has focused on increasingly emphasized the impact of the human microbiota on individual health. A recent article published in Nature Communications suggested a potential link between gut microbiota diversity and the severity of depression, suggesting that dysregulated gut microbiota may influence depressive symptoms by disrupting the immune system and modulating inflammatory factors [4]. However, it should be noted that this study utilized a mouse model; thus, although these findings provide preliminary insights into the possible mechanisms underlying microbiota-depression interactions, direct extrapolation to humans is premature [4].

Apart from the extensively studied gut microbiome, the oral microbiome also plays an essential role within the microbial ecosystem, forming a significant microenvironment hosting between 500 billion to 1 trillion bacteria [5]. As the second-largest microbial community in the human body, the oral microbiome resides at the entry of the digestive system [6]. These oral microbial communities have prominent roles in immunological and physiological functions, including preventing pathogen invasion and growth, balancing anti-inflammatory and pro-inflammatory responses, maintaining immune system homeostasis, and detoxifying or processing environmental chemicals [7]. Research indicates a strong correlation between the oral microbiome and overall health; thus, changes in oral microbiome may be associated with cardiovascular, immune, endocrine, neurological, gastrointestinal disorders, as well as oral diseases [8]. According to the clinical research by Gao et al. [9], the diversity and richness of oral microbiome decrease during and after radiotherapy. Moreover, the oral microbiome is strongly related to various metabolic conditions, including obesity, hyperglycemia and diabetes [10–12].

Nevertheless, only a limited number of studies have focused on the relationship between depression and oral microbiome diversity. The exploration of the relationship between depression and oral microbiome from the perspective of oral microbiota represents a novel research direction. Although Liu et al*.* [4] demonstrated in their rodent model that gut microbiota alterations correlated with behavioral changes via the gut-brain axis, direct evidence of microbiota-driven depression causation in humans remains elusive. The oral-gut-brain axis concept recently introduced by Paudel et al*.* [13]. suggests that the gut and oral cavity can communicate mutually through their respective microbiota. Given the significant role of the oral microbiome, it is necessary to explore the connection between depression and oral microbiome diversity. Investigating this relationship could not only facilitate a more thorough understanding of the pathogenic mechanisms of depression but also contribute to the development of novel therapeutic targets or biomarkers.

A few studies have begun addressing this research gap, suggesting that oral microbial diversity may be associated with mood disorders [14, 15]. However, these studies are limited in both number and scope, frequently targeting specific populations or employing small sample sizes. Due to this literature gap, further research in more diverse and larger populations is needed to clarify the potential relationship between oral microbiome diversity and depression.

Based on the data presented by the NHANES (National Health and Nutrition Examination Survey), the goal of this research is to address this gap by investigating the relationship between depression and oral microbiome diversity in a nationally representative sample.

## Methods

### Data and sample sources

**Fig. 1:**
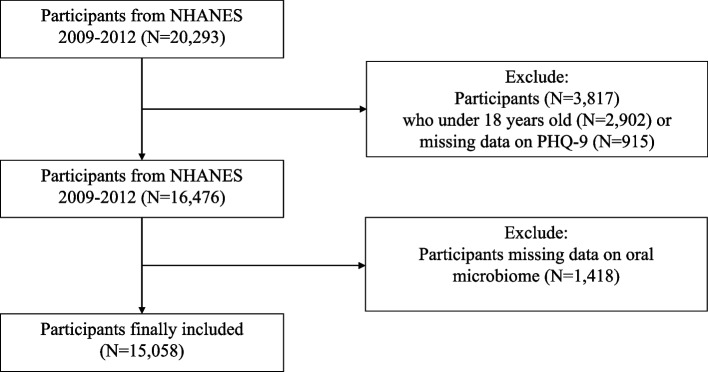
Flowchart of the sample selection from NHANES 2009–2012

The data involved in this research were partly collected through the NHANES (National Health and Nutrition Examination Survey) carried out by the CDC (Centers for Disease Control and Prevention). In addition, some data were accessed through the NHANES website (https://www.cdc.gov/nchs/nhanes.htm). The NHANES survey protocol was approved by the National Center for Health Statistics (NCHS) Ethics Review Committee, with all participants providing written consent. This research is not a clinical trial and the clinical trial number is not applicable. In this research, NHANES data covered the periods of 2009–2010 and 2011–2012; and the inclusion criteria were: (1) participants aged 18 years or older; (2) completed the PHQ-9 questionnaire; and (3) participated in the oral microbiome data collection. Initially, 20,293 individuals participated in the survey. After excluding participants younger than 18 years old (2,902 individuals), those with incomplete PHQ-9 data (915 individuals), and those with incomplete oral microbiome data (1,418 individuals), 15,058 eligible individuals were in the final analysis as shown in Fig. 1.

### Outcome variable

#### Oral microbiome diversity

Oral rinse samples collected through NHANES during the period of 2009–2010 and 2011–2012 were used in this research to test oral microbiome. Details concerning the procedures of DNA extraction, sequencing and bioinformatics analysis can be found on the NHANES website [16]. In brief, participants provided oral rinse samples for DNA extraction, followed by 16S rRNA PCR amplification and sequencing. The DADA2 and QIIME software were used to process raw sequence files, so as to assign taxonomic classifications and generate ASVs (amplicon sequence variants).

The alpha diversity is used to assess microbiome variety within an individual participant, with a focus on community evenness and richness. The NHANES data offered four metrics for this goal: the Shannon–Wiener Index, the Simpson Index, Faith’s Phylogenetic Diversity and observed ASVs. In this research, observed ASVs were emphasized, which was a metric directly reflecting the number of unique microbial taxa in a sample and commonly used for examining the diversity shifts and comparing sample richness between various exposure groups. Saturation of observed ASVs occurred at a rarefaction value of 10,000 [16], and this value was utilized for analysis.

Beta diversity, contrastingly, assesses the differences in microbiome composition between participants by using pairwise dissimilarity. As a popular beta-diversity analysis method, Bray–Curtis dissimilarity [17], chosen for this research, takes species abundance and presence into account and highlights differences in total species abundance between samples. This robust metric is widely applied to compare the compositions of microbial community across different treatments and evaluate the differences or similarities in communities.

#### Depression

The PHQ-9 (9-item Patient Health Questionnaire) was adopted to evaluate depressive symptoms. According to the Diagnostic and Statistical Manual of Mental Disorders (4 th edition) [18], PHQ-9 is an effective tool designed to evaluate a total of nine diagnostic criteria of major depression. It has been developed as a concise and effective screening tool for identifying major depression in clinical and research settings. The PHQ-9 questionnaire evaluates the impact of individual symptoms on the respondent over the preceding two-week period, utilizing a rating scale from 0 (no presence of the symptom) to 3 (symptom occurs nearly every day) [18]. The range of the PHQ-9 score is 0–27, and a higher score indicates more serious symptoms of depression [18]. This study used a standardized PHQ-9 score that met or exceeded ten as the criterion for clinically significant manifestations of depression [18]. Participants'PHQ-9 scores in this study were divided into two groups: < 10 (no depression) and ≥ 10 (depression) [19]. It has been reported that the specificity and sensitivity of PHQ-9 in determining major depression was 88% [18].

### Covariables

To increase the precision of analysis results and control for underlying confounding factors, this research included a variety of covariables. Variables, assumed or known to be linked with the oral microbiome, depression, or both, were selected from the NHANES data files [20–23]. These covariables comprised age (coded as a continuous variable), education level (college or above, high school, below high school), gender (female or male), and race (non-Hispanic Black, non-Hispanic White, Mexican American, Hispanic, etc.). Besides, the BMI (body mass index), categorized as ≥ 30 kg/m^2^ (obesity), 25–30 kg/m^2^ (overweight), and ≤ 25 kg/m^2^ (normal weight), and waist circumference (coded as a continuous variable) were also included in this research. The covariables also included sleep disorders categorized as “yes” or “no”, answering the question “whether you have once told a doctor about your sleeplessness”. Smoking was categorized into three groups: No, Former, and current. “No” refers to never smokers, defined as individuals who have never smoked. “Former” indicates former smokers, defined as individuals who have previously smoked more than 100 cigarettes but have since quit. “current” represents current smokers, defined as participants aged 20 years or older who reported smoking occasionally or daily, or having smoked more than 100 cigarettes in their lifetime [24, 25]. Excessive alcohol consumption was defined as consuming five or more drinks per day on average in the past 12 months prior to the interview [26]. Diabetes status was classified as ‘yes’ if participants had fasting glucose levels ≥ 7.0 mmol/L or reported having been diagnosed by a doctor; otherwise, it was classified as ‘no’. Hypertension status was similarly classified as ‘yes’ if there was a self-reported diagnosis by a doctor; otherwise, it was classified as ‘no’. Oral treatment was defined as having undergone gum disease treatments such as scaling and root planning, also referred to as ‘deep cleaning’.

### Statistical analysis

The sample analysis adhered to NHANES weighting guidelines. Weighted linear regression models and the mean ± standard deviation was employed to compare and describe the continuous variables, respectively. Weighted chi-square tests (for categorical variables) or rank-sum tests (as appropriate), along with weighted percentages, were used to compare and describe categorical variables. The observed number of ASVs was analyzed both as a categorical variable (quartiles) and as a continuous variable. Differences between groups for continuous and categorical variables were evaluated using weighted t-tests and weighted chi-square tests, respectively.

The association between depression and alpha diversity was assessed using a weighted multiple logistic regression model, with depression (PHQ-9 ≥ 10) as the binary outcome variable and the number of ASVs as the main predictor. In terms of data presentation, when ASVs were included as continuous variables in the weighted multiple logistic regression model, a log₁₀ transformation was applied where appropriate to enhance interpretability and improve statistical robustness. Three models were utilized, namely, Model 1 (unadjusted), Model 2 (adjusted for race, gender, and age), and Model 3 (fully adjusted for all covariates). To evaluate the nonlinear relationship between the number of ASVs observed and depression, smoothing curve fitting and the weighted generalized additive models (GAMs) were adopted. A turning point was ascertained by analyzing two-piecewise linear regression models based on the smoothing curve. The association between depression and the number of ASVs across different covariate subgroups was examined through subgroup analyses.

Overall compositional differences in the microbiome between participants based on Bray–Curtis beta-diversity were analyzed and visualized through non-metric multidimensional scaling (NMDS), which was a robust and unrestricted ordination method [27]. The statistical significance of differences between depression and non-depression groups was tested using Analysis of Similarity (ANOSIM) [28]. Empower Stats (version 4.2), SAS software (version 9.4), and R software (version 3.4.3) were utilized to perform statistical analyses, with statistical significance set at a two-tailed P-value < 0.05.

## Results

### Sample characteristics

There were 15,018 participants in this study, with 7,370 (49.51%) participants being women, and 946 participants diagnosed with depression. The mean age of the participants was 42.2 years (SD = 15.20 years). With respect to ethnicity and race, the participants were composed of 2,519 Mexican Americans, 1,556 Hispanics, 5,717 non-Hispanic Whites, 3,580 non-Hispanic Blacks, and 1,646 individuals from other ethnic groups. Participants with depression, compared to those without depression, tended to be younger than 60 years, non-Hispanic White, female, obese, smokers, and more likely to have hypertension or diabetes. Significant differences were observed between the two groups across all included variables (all *P* < 0.001). Table 1 provided more details on the characteristics of the participants.

**Table 1:** Weighted Sample characteristics classified by depression status among U.S. adults (*N* = 15,018)

Characteristics	Participant group, N (%)			*P*
	All 15,018	Non-depression 13,530 (89.9)	Depression 1,488 (10.1)	
Age (years)	42.2 ± 15.20	42.1 ± 15.33	43.55 ± 14.32	< 0.001
Age				0.024
< 60	12,232 (85.89)	10,988 (85.39)	1,244 (91.36)	
≥ 60	2,786 (14.11)	2,542 (14.61)	244 (8.64)	
Gender, (%)				< 0.001
Male	7,648 (50.49)	7,106 (51.65)	542 (37.77)	
Female	7,370 (49.51)	6,424 (48.35)	946 (62.23)	
Race/ethnicity, (%)				< 0.001
Mexican American	2,519 (10.19)	2,270 (10.05)	248 (11.63)	
Hispanic	1,556 (5.25)	1,356 (5.14)	200 (6.50)	
Non-Hispanic White	5,717 (64.89)	5,134 (65.44)	584 (58.88)	
Non-Hispanic Black	3,580 (12.49)	3,232 (12.09)	348 (16.84)	
Other	1,646 (7.19)	1,538 (7.29)	108 (6.15)	
Education level, (%)				< 0.001
< high school	3,519(16.44)	3,003 (16.21)	535 (30.74)	
High school	3,293 (22.23)	2,964 (22.14)	329 (23.24)	
College	4,579 (30.95)	4,101 (30.73)	467 (33.29)	
> College	3,627 (29.38)	3,462 (30.92)	157 (12.73)	
Family PIR	2.42 ± 1.68	2.52 ± 1.68	1.53 ± 1.34	< 0.001
BMI, (%)				< 0.001
≤ 25	4,683 (32.12)	4,280 (32.38)	401 (29.32)	
25 to 30	4,747 (32.09)	4,395 (32.72)	353 (25.20)	
≥ 30	5,588 (35.78)	4,855 (34.89)	734 (45.48)	
Waist circumference, (cm)	97.98 ± 16.70 (range 59.7 to 179)	97.61 ± 16.49	101.39 ± 18.16	< 0.001
Sleep disorder, (%)				< 0.001
Yes	1,202 (7.75)	836 (6.31)	366 (23.45)	
No	13,816 (92.25)	12,694 (93.69)	1,122 (76.55)	
Oral treatment, (%)				0.490
Yes	3,610 (20.67)	3,239 (20.37)	370 (24.06)	
No	11,408 (79.33)	10,291 (79.63)	1,118 (75.94)	
Excessive alcohol, (%)				< 0.001
Yes	2,925 (17.25)	2,545 (16.91)	390 (21.41)	
No	12,092 (82.75)	10,985 (83.09)	1,098 (78.59)	
Smoke, (%)				< 0.001
No	8,434 (55.46)	7,842 (56.92)	598 (39.71)	
Former	3,028 (22.16)	2,764 (22.73)	265 (15.93)	
Now	3,556 (22.38)	2,924 (20.34)	625 (44.36)	
Diabetes, (%)				< 0.001
Yes	1,738 (8.57)	1,448 (8.10)	290 (13.66)	
No	13,730 (91.43)	12,082 (91.90)	1,198 (86.34)	
Hypertension, (%)				< 0.001
Yes	4,218 (24.54)	3,614 (23.47)	604 (36.28)	
No	10,800 (75.46)	9,916 (76.53)	884 (63.72)	
ASVs	131.30 ± 43.78 (range 10.2 to 348.2)	131.72 ± 43.38	127.56 ± 47.13	0.002
LogASVs	2.09 ± 0.16	2.09 ± 0.16	2.07 ± 0.19	0.002

This research presented continuous variables as Mean ± SD, and the weighted linear regression model was used to calculate *P*-values (P). Besides, the categorical variables were presented in percentage (%), with the P calculated through the weighted chi-square test

*Abbreviations*: *PIR* poverty income ratio, *BMI* body mass index, *ASVs* Amplicon Sequence Variants, *N* Number

### Association between depression and alpha diversity of oral microbiome

#### Association between depression and the number of observed ASVs

This study found a significant negative association between the number of observed ASVs and the risk of depression. In the unadjusted model, a higher number of observed ASVs was significantly associated with a lower risk of depression (OR = 0.76, 95% CI: 0.63 to 0.87, *p* < 0.001). This association remained significant even after adjusting for all confounding variables (OR = 0.85, 95% CI: 0.69 to 0.99, *p* < 0.01).

When the number of observed ASVs was categorized into quartiles, all models consistently demonstrated a significant inverse relationship between increasing ASV counts and the reduced risk of depression. In the fully adjusted model, participants in the highest quartile (Q4) had a significantly lower risk of depression compared to those in the lowest quartile (Q1) (OR = 0.81, 95% CI: 0.68 to 0.97, *p* < 0.01) (Table 2).

**Table 2:** Correlation between the Depression and the number of observed ASVs

	Model 1 OR (95% CI)	Model 2 OR (95% CI)	Model 3 OR (95% CI)
Number of ASVs Continuous	0.76 (0.63, 0.87) ***	0.87 (0.72, 1.01)	0.85 (0.69, 0.99)**
Quartile 1	1 (ref)	1 (ref)	1 (ref)
Quartile 2	0.71 (0.58, 0.81) ***	0.72 (0.61, 0.85) ***	0.76 (0.62, 0.95) **
Quartile 3	0.84 (0.71, 0.97) *	0.89 (0.78, 1.05)	0.89 (0.75, 1.07)
Quartile 4	0.79 (0.68, 0.91) **	0.89 (0.77, 1.05)	0.81 (0.68, 0.97) **
P for tend	< 0.001	< 0.001	< 0.001

No covariates in Model 1 were adjusted. Race, gender and age in Model 2 were adjusted. Race, gender, age, education level, Family PIR, BMI, waist circumference, sleep disorder, oral treatment, excessive alcohol, alcohol, smoke, diabetes, and hypertension in Model 3 were adjusted

*Abbreviations*: *PIR* the ratio of income to poverty, *BMI* body mass index, *ASVs* Amplicon Sequence Variants represent, *Q* quartile. ***/**/* indicates *p* < 0.001, 0.01 and 0.05, respectively

#### Nonlinear relationship between LogASVs and depression

**Fig. 2:**
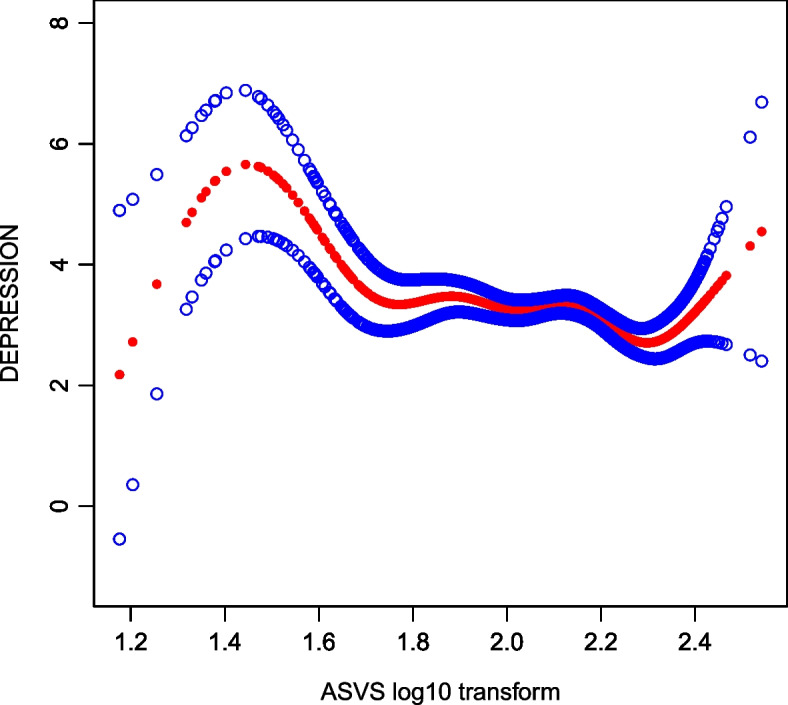
Linear relationship between depression and LogASVs based on the generalized additive model

In this study, smoothing curve fitting was used to examine the nonlinear relationship between LogASVs and depression scores based on the PHQ-9 scale (Fig. 2). The results indicated a significant nonlinear association. Threshold effect analysis (Table 3) revealed an inflection point at 2.32 for LogASVs (log-likelihood ratio test *p* = 0.011). When LogASVs were below 2.32, each unit increase in LogASVs was associated with a 1.48-point reduction in the PHQ-9 depression score. However, when LogASVs exceeded 2.32, each additional unit increase was associated with a significant rise in depression scores.

**Table 3:** Analysis on Threshold Effect of LogASVs on Depression based on two-piecewise linear regression model

Outcome	Depression [β (95% CI)], *P*
Fitting by the standard linear model	−1.20 (−1.83, −0.58), < 0.001
Fitting by the two-piecewise linear model	
Inflection point (K)	2.32
< K-segment effect	−1.48 (−2.13, −0.82), < 0.001
> K-segment effect	8.27 (0.92, 15.61), 0.028
Log likelihood ratio	0.011

Race, gender, age, education level, Family PIR, BMI, waist circumference, sleep disorder, oral treatment, excessive alcohol, alcohol, smoke, diabetes, and hypertension were adjusted

*Abbreviations*: *PIR* the ratio of income to poverty, *BMI* body mass index, *ASVs* Amplicon Sequence Variants represent

### Association between depression and beta diversity of oral microbiome

**Fig. 3:**
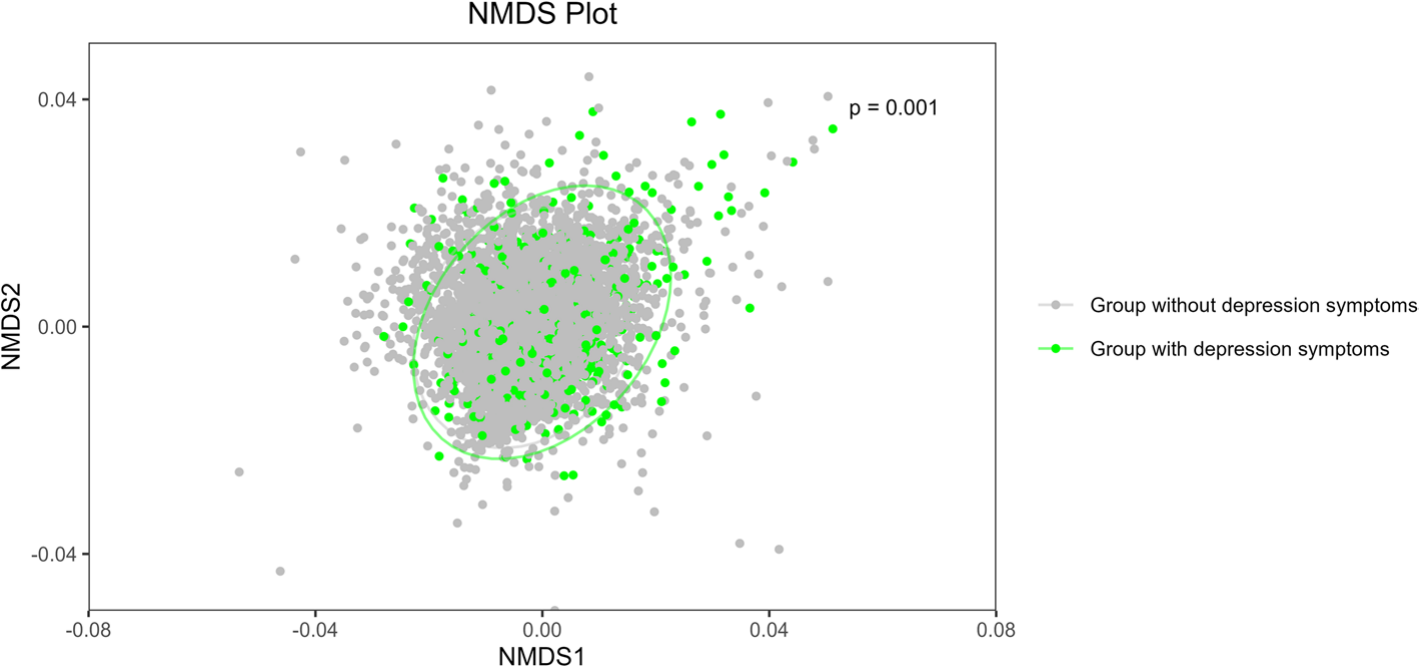
The NMDS Plot represents the Bray–Curtis dissimilarity of groups with and without depression symptoms

The NMDS plot in this research illustrated the separation of groups with and without depressive symptoms, based on Bray–Curtis distances. The analysis showed that groups with and without depression symptoms differed significantly in the multivariate ordination space (*p* = 0.001) (Fig. 3).

### Subgroups analysis

This study categorized participants according to gender, age, ethnicity, excessive alcohol consumption, smoking and oral treatment to determine the relationship between depression and the number of LogASV observed. Subgroup results found statistically significant differences between subgroups of excessive alcohol, smoke, and oral therapy. The results of the interaction test showed that the *P* for interaction for the subgroups by race and gender were < 0.001, indicating that gender and race had a significant moderating effect on the relationship between depression and the number of LogASVs (Table 4).

**Table 4:** Subgroup analysis on the correlation between depression and the number of observed ASVs

Subgroup	Depression [OR (95%CI)], *P*	*P* for interaction
Sex		< 0.001
Male	0.14 (0.06, 0.30), < 0.001	
Female	0.86 (0.54, 2.37), 0.734	
Age		0.216
< 60 years	0.51 (0.28, 0.92), 0.025	
≥ 60 years	0.21 (0.06, 0.75), 0.017	
Race/ethnicity		0.002
Mexican American	0.81 (0.17, 3.82), 0.787	
Hispanic	0.89 (0.13, 6.13), 0.906	
Non-Hispanic White	0.16 (0.07, 0.35), < 0.001	
Non-Hispanic Black	3.00 (0.81, 11.11), 0.100	
Others	0.10 (0.01, 1.48), 0.094	
Education level		0.169
< high school	1.47 (0.51, 4.27) 0.476	
High school	0.38 (0.20, 0.74) 0.004	
College	0.41 (0.19, 0.90) 0.025	
> College	0.52 (0.25, 1.08) 0.080	
BMI		0.265
< 24.9 kg/m^2^	0.86 (0.40, 1.83) 0.695	
25–29.9 kg/m^2^	0.37 (0.18, 0.76) 0.007	
≥ 30 kg/m^2^	0.48 (0.28, 0.82) 0.008	
Diabetes		0.091
Yes	0.26 (0.11, 0.61) 0.002	
No	0.59 (0.39, 0.90) 0.014	
Sleep disorder		0.066
Yes	1.08 (0.46, 2.54) 0.854	
No	0.45 (0.29, 0.68) < 0.001	
Hypertension		0.963
Yes	0.47 (0.27, 0.82) 0.008	
No	0.48 (0.30, 0.77) 0.003	
Excessive alcohol		0.139
Yes	0.24 (0.09, 0.63), 0.004	
No	0.57 (0.30, 1.07), 0.080	
Smoke		0.277
No	0.72 (0.26, 2.02), 0.536	
Former	0.50 (0.16, 1.59), 0.240	
Now	0.26 (0.12, 0.57), < 0.001	
Oral treatment		0.144
Yes	1.03 (0.27, 3.95), 0.963	
No	0.35 (0.19, 0.63), < 0.001	

## Discussion

In this population-based study, oral microbiome diversity was found to be negatively associated with depressive symptoms. Specifically, alpha diversity of the oral microbiome was negatively correlated with depression symptoms, even after adjusting for potential confounding variables. Threshold effect analysis further revealed that that this relationship was nonlinear. Subgroup analysis indicated that smoking, excessive alcohol consumption, and oral treatment influenced the association between oral microbiology and depression, with interaction effects observed across gender and racial groups. Additionally, beta diversity of oral microbiome showed significant differences between participants without and with depressive symptoms (*P* = 0.001). In this cross-sectional analysis, we observed an association between oral microbiome diversity and depressive symptoms in a nationally representative cohort. The directionality of this relationship remains uncertain. It is possible that the oral microbiome influences depressive symptoms through immune modulation, inflammation, or the oral–gut–brain axis. Conversely, depressive symptoms may lead to behavioral changes—such as altered diet, impaired oral hygiene, and increased substance use—that subsequently affect the oral microbial ecosystem. Given the cross-sectional design, we are unable to infer causality. Future longitudinal and mechanistic studies are warranted to clarify the direction and underlying pathways of this relationship.

As far as we know, this research is among the first to investigate the association between depression and diversity of oral microbiota. Several potential mechanisms have been hypothesized to link oral microbiota with depression. First, oral microbiota may contribute to the pathogenesis of depression through the"oral-gut axis."Compared to the gut microbiota, the oral microbiota exhibits unique α-diversity characteristics and genus-level compositional features [29]. This niche-specific ecological trait enables oral microbes, during their migration to the gut, to significantly alter the composition of the gut microbiota through mechanisms such as competitive colonization and metabolite exchange. For example, gut colonization by Fusobacterium nucleatum (a pathogen typically restricted to the oral cavity) can lead to decreased abundance of Bacteroidetes and increased proportions of Firmicutes [29, 30]. Such taxonomic shifts in microbial composition are significantly correlated with depression-associated gut barrier impairment [31]. Within the bidirectional regulatory network of the"gut-brain axis,"this cross-niche microbial interaction may mediate cortisol release, glucocorticoid receptor expression, and HPA axis activation via the enteric nervous system [32, 33], ultimately establishing a pathological link to depression. Second, neuroimmune responses induced by oral microbiota may be a potential pathway affecting depression. Porphyromonas gingivalis (P. gingivalis) invasion and colonization form dental plaque, which is a primary cause of periodontitis [34, 35]. This pathogen and its virulence factors not only remodel microbial communities and accelerate dysbiosis but also spread systemically, inducing low-grade neuroinflammation [36, 37]. For instance, microglia can be activated by P. gingivalis ATCC 33277, resulting in releasing pro-inflammatory factors, like CRP and IL-1OR [38]. These factors cross the blood–brain barrier, inducing neuroinflammation, causing hippocampal neuron damage, and increasing the odds of depression [39, 40]. Third, the metabolic products of oral microbiota regulate neurotransmitters to modulate depression. Oral microbial metabolism of short-chain fatty acids (SCFAs) in plasma is involved in regulating the metabolism of neurolipids and inflammatory factors, balancing neurotransmitters, and exerting an attenuated anti-inflammatory response [41, 42]. Jiang Wenxia et al*.* found that SCFA dysregulation significantly altered the metabolism of neurotransmitters such as phosphatidylcholine (PC) and triglycerides (TG) in the prefrontal cortex, thereby inducing depressive-like behavior [43]. In summary, oral microbiota participates in the development and progression of depression through pathways involving the enteric nervous system, neuroimmune responses, and neurotransmitter regulation. In this study, we present the first evidence of an association between oral microbiome diversity and depression, derived from a cross-sectional analysis utilizing data from a nationally representative cohort.

As highlighted by Dumitrescu [44], the relationship between oral microbiota and depression may be bidirectional. Depression may influence the composition and health of the oral microbiota through various pathways, including physiological mechanisms, lifestyle habits, and medication use. One of the main features of depression development is the dysregulation of the HPA (hypothalamic–pituitary–adrenal) axis [45], which is usually linked with the increasing neuroinflammatory activity [46, 45]. This heightened inflammatory state can alter the composition of the oral microbiota in individuals with conditions such as periodontitis. In addition, individuals with depression are more likely to engage in unhealthy lifestyle habits, which not only exacerbate their physical and mental health conditions but also have a considerable impact on the oral microbiome. Studies have shown that depression is associated with higher rates of alcohol consumption and smoking—behaviors that significantly alter the composition and stability of the oral microbial community. Smoking, for example, reduces microbial diversity and increases the abundance of pathogenic bacteria such as Porphyromonas gingivalis, thereby promoting periodontal inflammation and disease progression. Alcohol consumption may disrupt the pH balance and immune environment of the oral cavity, creating favorable conditions for the overgrowth of anaerobic bacteria [47]. Furthermore, individuals with depression often adopt poor dietary habits, such as high intake of sugar and fat [48], which provide abundant substrates for cariogenic bacteria like Streptococcus mutans, leading to increased plaque formation, higher risk of dental caries, and altered microbial composition [49].

Depressive symptoms may also impair self-care motivation, resulting in inadequate oral hygiene practices such as infrequent tooth brushing and irregular dental visits. These behavioral patterns accelerate plaque accumulation and disrupt the ecological balance of the oral microbiota [50]. Collectively, these factors underscore the multifaceted and dynamic relationship between depression-related behaviors and oral microbial dysbiosis. In terms of pharmacological effects, although certain antidepressants (e.g., fluoxetine) may improve periodontal health through anti-inflammatory mechanisms [51], it is crucial to note that most psychotropic medications (such as tricyclic antidepressants and antipsychotics) exhibit anticholinergic side effects [52, 53]. These effects can lead to reduced salivary secretion (xerostomia), thereby significantly altering the oral microenvironment [54]. Thus, medications not only alleviate depressive symptoms but also have a regulatory effect on oral health [55]. The current study establishes an association between the oral microbiota and depression in a cross-sectional manner. While it does not clarify the directionality of this relationship, the potential for a bidirectional interaction is acknowledged. The precise nature of their interaction and the causal relationship between them require further investigation. It is important to note that the present study is a cross-sectional analysis, which allows us to explore the association between the oral microbiome and depression in a nationally representative population. However, due to the limitations of the study design, causal relationships between these two variables cannot be established. Therefore, future longitudinal cohort studies are necessary to clarify the directionality of this relationship and to further investigate the underlying mechanisms.

This research identified a nonlinear relationship between depression and diversity of oral microbiota, which may be closely related to the maintenance of microbial ecological niche balance. Maintaining microbial ecological balance is crucial for generating host–microbiota feedback effects, as disturbances in this balance are often accompanied by metabolic disorders [56]. Within a certain range of microbial richness, an increase in oral microbiota alpha-diversity is associated with a significant decrease in depression. However, this trend disappears once alpha-diversity reaches a specific threshold. One possible explanation is that moderate alpha-diversity helps stabilize the microbial ecosystem, thereby inhibiting the growth of pathogenic bacteria [5]. This balance may be achieved through competitive exclusion and increased resource utilization efficiency, which positively influence the body's immune response [57]. Conversely, a continuous increase in alpha-diversity may lead to an upward trend in depression, potentially due to an overgrowth of oral microbiota causing an ecological imbalance. This imbalance, marked by a disruption between beneficial bacteria and pathogens or a lack of immune-regulating symbiotic bacteria, could trigger or exacerbate systemic inflammatory responses, ultimately affecting mental health [5, 57].

In the subgroups characterized by excessive alcohol consumption, smoking, or oral treatment, significant differences were observed in the association between oral microbiota alpha diversity and depression. Several potential mechanisms may underlie these differences. Excessive alcohol consumption has been shown to disrupt oral microbiota homeostasis [58], a finding consistent with results from large-scale population-based studies [47]. Ethanol inhibits the proliferation of acid-producing bacteria such as Lactobacillus, leading to an imbalance between Bacteroidetes and Firmicutes, which ultimately results in a marked reduction in alpha diversity [59]. Similarly, smoking significantly reduces oral microbiota alpha diversity and exacerbates ecological dysbiosis [60]. Nicotine impairs bacterial biofilm integrity and inhibits key metabolic enzymes, such as NADH dehydrogenase, selectively depleting sensitive commensal bacteria like *Actinomyces*, while increasing the relative abundance of antibiotic-resistant pathogens such as *Streptococcus mutans* [60, 61]. *S. mutans* is a primary cariogenic organism frequently found in patients with both depression and dental caries [62]. Furthermore, Streptococcus species can penetrate the blood–brain barrier [63] and promote the formation of a pro-inflammatory microenvironment in the central nervous system through microglial activation [64], thereby increasing susceptibility to depression. In addition, oral treatments—particularly periodontal therapy—may temporarily reduce alpha diversity by eliminating pathogenic bacteria. Oral treatments such as scaling, root planning, and other"deep cleansing"may reduce periodontal pathogens in the short term, decreasing the diversity of ASVs and weakening their anti-inflammatory function. This transient disturbance weakens the microbiota’s homeostatic capacity, manifesting as decreased probiotic abundance or impaired metabolic activity. In contrast, the natural oral microbiota may modulate the immune system through metabolites (e.g., short-chain fatty acids) and inhibit the transmission of peripheral inflammatory factors (e.g., IL-6) to the brain, thereby reducing the risk of depression. Notably, metabolic by-products of commensal oral microbes, such as butyrate, may reach the intestine and stimulate L-cells in the intestinal epithelium to secrete glucagon-like peptide-1 (GLP-1) [65]. GLP-1 may suppress the transmission of peripheral pro-inflammatory cytokines, such as IL-6, to the brain via the vagus nerve–nucleus tractus solitarius pathway, thereby contributing to a microbiota–neuroimmune barrier [66]. However, excessive oral hygiene practices may disrupt this balance, induce local or systemic inflammation, and consequently elevate the risk of depression. In conclusion, specific behaviors such as alcohol use and smoking, along with oral treatment interventions, may significantly influence the relationship between oral microbiota and depression. Nevertheless, further studies are needed to determine causal relationships and clarify the underlying mechanisms.

Notably, subgroup analyses not only revealed the moderating role of behavioral factors, but also highlighted the complex influence of demographic characteristics on the association between alpha diversity and depression. Gender differences significantly moderated the relationship between the number of ASVs and depression (*P* for interaction < 0.001), and alpha diversity was significantly negatively associated with depression in men. Interaction tests for racial differences further indicated that ethnicity significantly influences the relationship between oral microbiota and depression, with this variability being attributed to a range of factors. Recent research indicates that racial differences in health and disease extend beyond biology, involving a complex interplay of social, biological, behavioral, environmental, and societal influences [67]. Due to the combined effects of environmental and genetic factors, the relationship between depression and diversity of oral microbiome varies across different ethnic groups. Although microbial composition within individuals tends to be relatively stable, considerable heterogeneity exists between individuals [68, 69]. Many disorders exhibit significant differences in prevalence across racial groups; For instance, compared with non-Hispanic whites, Native Americans, Hispanics and African Americans face higher prevalence of type 2 diabetes. Even monozygotic twins, who share identical or nearly identical genotypes, show differences in physical and mental health outcomes due to environmental influences [70]. Additionally, sociocultural factors, such as traditional dietary habits, play a crucial role in shaping the oral microbiome. According to relevant research, the odds of depression will be reduced with diets rich in high-protein foods, grains, vegetables and fruits [71, 72]. Meanwhile, factors such as racial discrimination, sexism, mental health stigma, and disparities in social support have become increasingly important in explaining modern health disparities [71]. These factors indirectly affect the oral microbiota by influencing lifestyle and psychological states. In order to develop personalized intervention strategies and understand the pathogenesis of depression, it is essential to carry out further research on the mechanisms underlying racial and gender differences.

In spite of the important findings of this research, several limitations should be acknowledged. First, depression was assessed using the PHQ-9 at a single time point, which reflects participants’ current depressive symptoms (state) rather than lifetime or chronic depression (trait). This cross-sectional design does not allow differentiation between transient and persistent depressive symptoms, nor does it clarify whether alterations in the oral microbiome are a cause or consequence of depression. Longitudinal studies are therefore needed to clarify the temporal sequence and potential bidirectional relationship between depression and oral microbiota diversity. Second, the inherent limitations of the cross-sectional design further contribute to the ambiguity regarding the causal direction between dysbiosis and depressive symptoms. Future longitudinal studies should incorporate additional variables related to the course of depression, such as episode frequency and duration, to better capture its clinical trajectory. Third, although this study controlled for several potential confounders, the possibility of residual confounding cannot be ruled out. For instance, chronic physical conditions and medication use may influence both the oral microbiota and depressive symptoms, potentially biasing the results.

Therefore, further prospective studies are warranted to investigate the underlying mechanisms linking depressive symptoms with oral microbiota, building upon the current findings. Nevertheless, this study contributes meaningfully to the growing body of literature on the association between depression and oral microbiota diversity.

## Conclusion

In conclusion, this study elucidated the association between oral microbiota diversity and depression, addressing a significant gap in the field by analyzing a large, nationally representative sample. Oral microbiota diversity was significantly negatively correlated with depression, and this correlation varied by race and gender. Additionally, this association changed when the ecological balance of oral microbiota was disrupted. These findings highlight that oral microbiota could serve as a predictive biomarker for depression. Furthermore, diagnostic and therapeutic approaches based on oral microbiota have great potential for the intervention and management of depressive disorders.

## Supplementary Information

Supplementary Material 1.

## Data Availability

The datasets used and analyzed during the current study are available from the corresponding author upon reasonable request. More information about the NHANES can be obtained at: http://www.cdc. gov/nhanes.

## Abbreviations

NHANES: National Health and Nutrition Examination Survey
PHQ-9: 9-Item Patient Health Questionnaire
ASVs: Amplicon Sequence Variants
16S rRNA: 16S Ribosomal Ribonucleic Acid
NMDS: Non-Metric Multidimensional Scaling
GAM: Generalized Additive Models
BMI: Body Mass Index
PIR: Poverty-Income Ratio
HPA Axis: Hypothalamic–Pituitary–Adrenal Axis
SCFAs: Short-Chain Fatty Acids
CRP: C-Reactive Protein
IL-1OR: Interleukin-1 Beta
PC: Phosphatidylcholine
TG: Triglycerides
FMT: Fecal microbiota transplantation
IRB: Institutional Review Board
